# A Moderate Increase in Ambient Temperature Influences The Structure and Hormonal Secretion of Adrenal Glands in Rats

**DOI:** 10.22074/cellj.2021.6827

**Published:** 2020-04-22

**Authors:** Florina Popovska-Perčinić, Milica Manojlović-Stojanoski, Lazo Pendovski, Suzana Dinevska Kjovkarovska, Biljana Miova, Jasmina Grubin, Verica Milošević, Vladimir Ajdžanović

**Affiliations:** 1. Department of Functional Morphology, Institute of Reproduction and Biomedicine, Faculty of Veterinary Medicine, Ss Cyril and Methodius University in Skopje, Lazar Pop Trajkov 5-7, Skopje, Republic of Macedonia; 2. Department of Cytology, Institute for Biological Research Siniša Stanković, University of Belgrade, 142 Despot Stefan Blvd., Belgrade, Republic of Serbia; 3. Department of Physiology and Biochemistry, Institute of Biology, Faculty of Natural Sciences and Mathematics, Ss Cyril and Methodius University in Skopje, Arhimedova 3, Skopje, Republic of Macedonia; 4. Ministry of Education, Science and Technological Development of the Republic of Serbia, Njegoševa 12, Belgrade, Republic of Serbia

**Keywords:** Adrenal Glands, Adrenocorticotropic Hormone, Corticosterone, Pituitary, Temperature

## Abstract

**Objective:**

As a consequence of global warming, the increase in the average annual temperature is observed, while the living organisms actively adapt to these changes. High environmental temperature initiates numerous physiological, autonomic, and behavioral responses, and activates the stress response. Thus, the aim of the study was to investigate effect of a moderate increase in ambient temperature on the activity of the hypothalamic-pituitary-adrenocortical (HPA) axis by determining histological changes in adrenal glands and hormonal levels in adult male rats. Material and Methods: In this experimental study, the morpho-functional state of adrenal glands was estimated by stereological evaluation of parameters, including the adrenal volume, adrenocortical cell/nuclear size and number, and the volume density of vascular tissues after four days of exposure to a moderate increase in ambient temperature of 35 ± 1˚C. Novelli histochemical and vascular endothelial growth factor (VEGF) immunohistochemical staining provided insight into the adrenal gland vascular network. Additionally, the adrenal levels of aldosterone, corticosterone, and pituitary adrenocorticotropic hormone (ACTH) were determined as crucial indicators of the hypothalamic-pituitary- adrenocortical (HPA) axis activity.

**Results:**

Prolonged exposure to a moderate increase in ambient temperature for four days resulted in a significant increase in ACTH level up to 24%, which altered adrenal glands both structurally and functionally. The adrenocortical volume and number of cells in all cortical zones were markedly increased (P<0.05). A statistically significant increase was shown in the level of aldosterone (16%) and corticosterone (25%) in serum levels of individuals.

**Conclusion:**

Increased activity of the HPA axis reflects the response to a moderate increase in ambient temperature during four days, showing the capacity of the HPA axis to adapt the organism to daily temperature changes.

## Introduction

The stress response initiates when afferent connections relay information from inner and outer environment to the hypothalamic nucleus paraventricularis (NPV), and more precisely to neurons that synthesize corticotropinreleasing hormone (CRH) and vasopressin (VP). Exposure to acute and chronic stress leads to the release of these hormones into the hypophyseal portal system in order to stimulate the production and secretion of adrenocorticotropic hormone (ACTH) from pituitary corticotrophs into the systemic circulation, which further regulates steroid production by the adrenal gland cortex (1). The adrenal cortex comprises of three concentric zones which are histologically and hormonally specific. Mineralocorticoids are synthesized in the small and ovoid cells of the zona glomerulosa (ZG) which is located beneath the capsule. Mineralocorticoids are controlled by kidneys and pituitary gland. The glucocorticoids are products of the significant part of the adrenal cortex named zona fasciculata (ZF) that consisted of steroidogenic cells arranged into radial lines and stimulated by pituitary ACTH. Zona reticularis (ZR), which is also regulated by pituitary hormones, is the most inner part of the adrenal cortex that produces androgens and other steroids.

Temperature is one of the most critical environmental factors that primarily determines the physiological responses. Any disturbance in homeostasis, such as temperature extremes instantly activates numerous physiological, autonomic, and behavioral responses. The hypothalamic-pituitary-adrenocortical (HPA) axis activation and an increase in circulating glucocorticoids represent the central part of the stress response (2). In stress conditions, such as high environmental temperatures, glucocorticoids affect numerous metabolic processes related to energy expenditure and storage. In order to mobilize energy, circulating glucocorticoids stimulate glycogenolysis, lipolysis, and proteolysis, which are vital processes for muscle and neural functions (3). The actions of glucocorticoids under the conditions of thermal stress are adaptive, as usual, and directed to increase energy availability, while the currently unnecessary/unessential physiological functions, such as reproduction or immunological defense, are slowed down or interrupted (4). By reducing metabolic heat production and increasing heat dissipation, organisms are able to efficiently cope with heat stress (5). Exposure to high environmental temperature causes the activation of the renin-angiotensinaldosterone system in order to sustain water and mineral homeostasis (6, 7). Aldosterone, secreted by adrenal gland cortex, incites reabsorption of ions, principally sodium, to avoid excessive loss of sodium and other electrolytes, indirectly influencing water retention or loss (7). Additionally, hypothalamic VP is another vital hormone that maintains water homeostasis during thermal stress. By acting in the kidney, VP stimulates reabsorption of water, thus regulating blood pressure, while together with aldosterone serves to another physiological mechanism, which is essential for survival in continuously changeable temperature conditions (8).

Keeping in mind fluctuations of the climate parameters, along with the results obtained from relevant official documents in the Western Balkan region, some high-risk changes, including intrusion of subtropical climate to the north, the increase of frequency and intensity of heatwaves, dry days, and extreme precipitation are anticipated. During the near future period, which is already happening (2016-2035), the mean annual temperature increase is expected to reach 0.5-1.0˚C, with a particular emphasis on the summer temperature (June-July-August) increase, that is higher than the mean annual up to 0.5-1.0˚C. Some calculations dramatically indicate that temperature for this season will exceed 5.0˚C increases at the end of the century, in comparison to the present climate (9). Usage of the long-term time series of mean annual air temperature confirms the elevated values for the Western Balkans region. The beginning of warming in this part of South-Eastern Europe during the last twentieth years is reported to start in a period between 1987 and 1997, mostly in 1988, while differences in the average mean annual air temperatures before and after warming are about 1˚C (10). Living organisms are very vulnerable to these changes that alter their safety, life quality, and distribution i.e., survival.

The animal thermal comfort zone is defined as the range of temperature in which animal metabolic and physiologic processes are stable and directed to the storage of carbohydrates, proteins, and fat (7). Our earlier findings showed increased activity of the pituitary corticotrophs as a result of an active resistance during four days of continuous exposure to elevated temperature (11). The consequences of 4-day exposure are characterized as short-term exposure, provoking metabolic, and physiological outcomes (12). Generally, the activation of the sympathoadrenomedullary system triggers the first reaction on the thermal stressor, and consequently, the activation of the HPA axis (13). In this work, the influence of a moderate increase in ambient temperature on the adrenal gland cortex, during a prolonged time period, was investigated in adult rats exposed to 35 ± 1℃ for four days. These temperature conditions exceed the upper-temperature comfort range and characterize the real conditions during summertime in the Western Balkan region, as already elaborated. In mammals (rats), as homoeothermic animals, the predictable response to increased environmental temperature starts with the HPA axis activation and may terminate with reduced growth, disturbances of vital functions, specific alterations in the central nervous system function, or extreme cases, lead to death (14). Thus, this study aimed to determine the histological changes and hormonal secretion of the adrenal glands, representing the activity of the HPA axis, and at the same time, being the indicators of the HPA axis disturbances under the conditions described earlier. Stereological measurements of the adrenal gland, as well as determination of adrenocortical aldosterone, corticosterone, and the pituitary ACTH circulation levels, provide respectable insights.

## Materials and Methods

### Animals and experimental protocol

In this experimental study, the experiments were conducted on adult male Wistar rats, weighing 260 g- 350 g. Animals were kept under standard conditions (12:12 hours light-dark cycle with free access to standard laboratory food and water). The animals were divided into two groups (7 animals per group): control and elevated temperature-exposed (experimental) group. The control group was kept at room temperature (20 ± 2℃), while the experimental group was continuously exposed for four consecutive days to moderately high ambient temperature (35 ± 1℃), in a special heat chamber with regulated air temperature and air humidity of 30-40%, as previously described (11). The specific temperature for the experimental group (35 ± 1℃) was chosen based on some previous investigations (15), established as a moderately high environmental temperature. Besides, the mode of continuous exposure was proposed in other studies (16). It should be mentioned that the climate region of South-Eastern Europe, which we belong to, is wellknown for having similar air temperatures during the summer months (15, 17). After four days of exposure, the animals were sacrificed by a laparotomic procedure under ether narcosis (Diethyl ether Stabil. G.R., Lach-Ner, s.r.o., 27711 Neratovice, Czech Republic). The sacrifice was performed between 8.00-9.00 AM. Subsequently, the blood samples were taken from arterial blood (a. dorsalis) and the plasma was frozen at -70℃ for the hormonal analysis, while the adrenal glands were excised, weighed, and prepared for the further histological analyses. All animal procedures were in accordance with the EU Directive 2010/63/EU and approved by the Ethical Committee for the Use of Laboratory Animals of the Institute for Biological Research Siniša Stanković University of Belgrade (approval no. 2-12/12).

### Histochemical and immunohistochemical staining

The adrenal glands were removed immediately after euthanasia, weighed, and fixed in 4% paraformaldehyde for 24 hours. After dehydration in ethanol with increasing concentrations, they were cleared in xylene and paraffin-embedded. For the histochemical staining and following the histological examinations and stereological measurements, adrenals were serially sectioned using a rotational microtome (RM 2125RT, Leica Microsystem, Wetzlar, Germany). The adrenal sections, at the thickness of 5 μm, were stained with hematoxylin-eosin and the Novelli method, which enables gaining insight into the tissue vascular profile and the measurement of vascular volume density (18). Hematoxylin-eosin staining procedure started with deparaffinization in xylene (2×5 minutes), rehydration in series of alcohol in decreasing gradient (100% ethanol, 96% ethanol, 70% ethanol; 5 minutes each), and continued with incubation in hematoxylin (3 minutes) followed by washing in tap water. The next step was eosin incubation (5 minutes) followed by brief immersion in 96% ethanol, dehydration in 100% ethanol (5 minutes) and incubation in xylene (2×5 minutes). Finally, the sections were mounted on Canada balsam. For the latter, after deparaffinization and rehydration, adrenal sections were incubated in heated 1N HCl (60˚C, 3 minutes), 1% acid fuchsine (30 seconds) and 1% light green (30 seconds), followed by washing in distilled water, and finally, dehydration and mounting were carried out. As a result, purple erythrocytes were clearly visible against the bright green background of the adrenal cortex. Digital visualization was obtained by a Leitz DM RB light microscope (Leica, Germany) with a DFC320 CCD camera (Leica Microsystems Ltd. Switzerland) and a DFC Twain Software (Leica, Germany).

Immunohistochemically labeled sections of the adrenal gland with vascular endothelial growth factor (VEGF), as an angiogenic peptide, provided insight into the capacity of the capillary network forming/branching. After deparaffinization and rehydration, the antigen retrieval procedure was performed by incubating sections in 0.01 M citrate buffer (pH=6.0) in a microwave (750 W) for 21 minutes (18). Endogenous peroxidase activity was blocked in 0.3% H 2O2 in methanol for 15 minutes, followed by blocking non-specific staining by 1 hour incubation with 10% normal swine serum (Dako, Denmark). Then, sections were incubated with rabbit polyclonal antiVEGF antibody (Abcam®, ab46154; Cambridge, MA, USA, 1:100) overnight at 4˚C. After washing in phosphate buffered saline (PBS), sections were incubated with secondary antibodies, polyclonal swine-anti-rabbit IgG/ HRP (Dako A/S, Glostrup, Denmark; 1:300) for 1 hour, at room temperature. Antibody localization was visualized by 0.05% 3,3-diaminobenzidine tetrahydrochloride (DAB) and counterstained with hematoxylin.

### Stereological measurements and morphometric analyses

The adrenal sections stained with hematoxylineosin were used for stereological measurements by a simple counting method (19, 20). Sections were examined under a light microscope with the aid of the Weibel multipurpose lattice M42 (42 points, 21 test lines) inserted into the ocular of the microscope. The volume of the adrenal gland, adrenal cortex volume, and volume of individual zones of the cortex [zona glomerulosa (ZG), zona fasciculate (ZF), zona reticularis (ZR)] were determined on serially sectioned adrenal glands. To prevent bias, from each adrenal sample, the first analyzed section was randomly chosen (choosing from 1st to 10th section), and then the measurements were performed on every10th section. Using ×100 magnification and mentioned M42 lattice, the total number of points falling on each adrenal cortex zone was counted. The volume of adrenal gland cortex and each zone of the cortex were calculated by multiplying the total number of test points by the area corresponding to one point and the thickness of the ten sections.

In order to measure the individual volume of adrenocortical cells and their nuclei for each adrenal gland zone, a single section containing the zona medullaris was selected, as a proxy of the central part of the gland. The 30-test areas of the ZG and 50-test areas of both the ZF and ZR were analyzed under a light microscope, at ×1000 magnification. The number of counts hitting cytoplasm and nuclei, as well as, the total cell number within the lattice M42 correspond to the size of individual cells or their nuclei, respectively. Earlier karyometric studies (19) estimated the shape coefficient to be 1.382 for the ZF, and 1.500 for the ZG. Since the adrenocortical cells are mononuclear, calculation of the numerical density (NV) (that corresponded to the number of cells per cubic millimeter) and Na (that corresponded to the number of cells in the plane of tissue sections) allowed the calculation of a single adrenocortical cell/nuclear volume.

The formula of Weibel (19) was used to determine the numerical density of the nuclei (Nv):

Nv=(kβ)×(Na32Vv12)

The cellular and nuclear volumes were calculated according to these formulas:

Vc=1Nv , and Vn=VvnNv

where V_V_n represents a nuclear volume density of the specific adrenocortical cell, providing information about the nuclei attendance, while N_V_ indicates a numerical density.

As the volumes of adrenocortical zones and volumes of single cells in each zone were calculated after conducted measurements, the number of adrenocortical cells for ZG, ZF, and ZR was calculated.

The estimation of the volume density was utilized to determine the percentage of vascular volume in the cortex. Image acquisition, morphometric assessment, and digital imaging were performed under a light microscope (Olympus BX-51, Olympus, Japan) and the newCAST stereological software package [Visiopharm Integrator System (VIS), version 5.3.1.1640, Visiopharm, Denmark]. Four central sections were analyzed per animal, with a spacing of 10 sections apart. The morphometric assessment was performed at a final magnification of ×490. The counting area was defined using a mask tool, while an interactive test grid with uniformly spaced test points for histomorphometric assessment was provided by the newCAST software.

Volume densities (V_V_) were calculated as the ratio of the number of points hitting the vascular compartment divided by the number of points hitting the analyzed area i.e., adrenal cortex:

Vv (%)=PpPt×100

where Pp represents counted points hitting the vascular tissue component and Pt is a total number of points of the test system hitting the adrenal cortex. The volume density of vascular tissues was calculated for each of the four sections, then for each animal, and at the end, the average value was calculated per group.

### Hormonal level measurements

For conducting the hormonal analysis, plasma and serum samples were used and stored at -70˚C until assay. The plasma levels of ACTH in both groups (experimental and control) were determined by the IMMULITE method (Diagnostic Products Corporation; Los Angeles, CA, USA) in duplicate samples within a single assay (18). The intra-assay coefficient of variation was 9.6%, while the analytical sensitivity of the assay was 9 pg/mL. Serum aldosterone concentrations were determined by enzyme immunoassay for direct quantitative determination (Aldosterone Elisa Kit, IBL, Germany) with intra-assay CV 7.4% and analytical sensitivity of 128.67 pg/mL. Serum corticosterone concentrations were measured without dilution by immunoassay in duplicate within single assays with an intra-assay CV of 8.0% (sensitivity of 171 pg/ml) (Corticosterone Immunoassay, R&D System Inc., USA)

### Statistical analysis

Data provided by stereological measurement and hormonal analysis were subjected to statistical analysis using the STATISTICA® version 5.0 (Stat Soft, Inc., USA) software. The stereological and hormonal data were evaluated by the Student’s t test. A P<0.05 level of confidence was assumed as the statistically significant result. All results are expressed as means for six animals per group.

## Results

### Body mass and adrenal gland weights

Exposure to the elevated temperature for four days leads to a significant reduction in body mass by 20%, as well as to a marked increase in absolute and relative adrenal gland weights by 16% and 25% respectively, compared with the control group (Table 1).

### Stereological parameters of the adrenal gland and hormonal analyses

**Fig.1:**
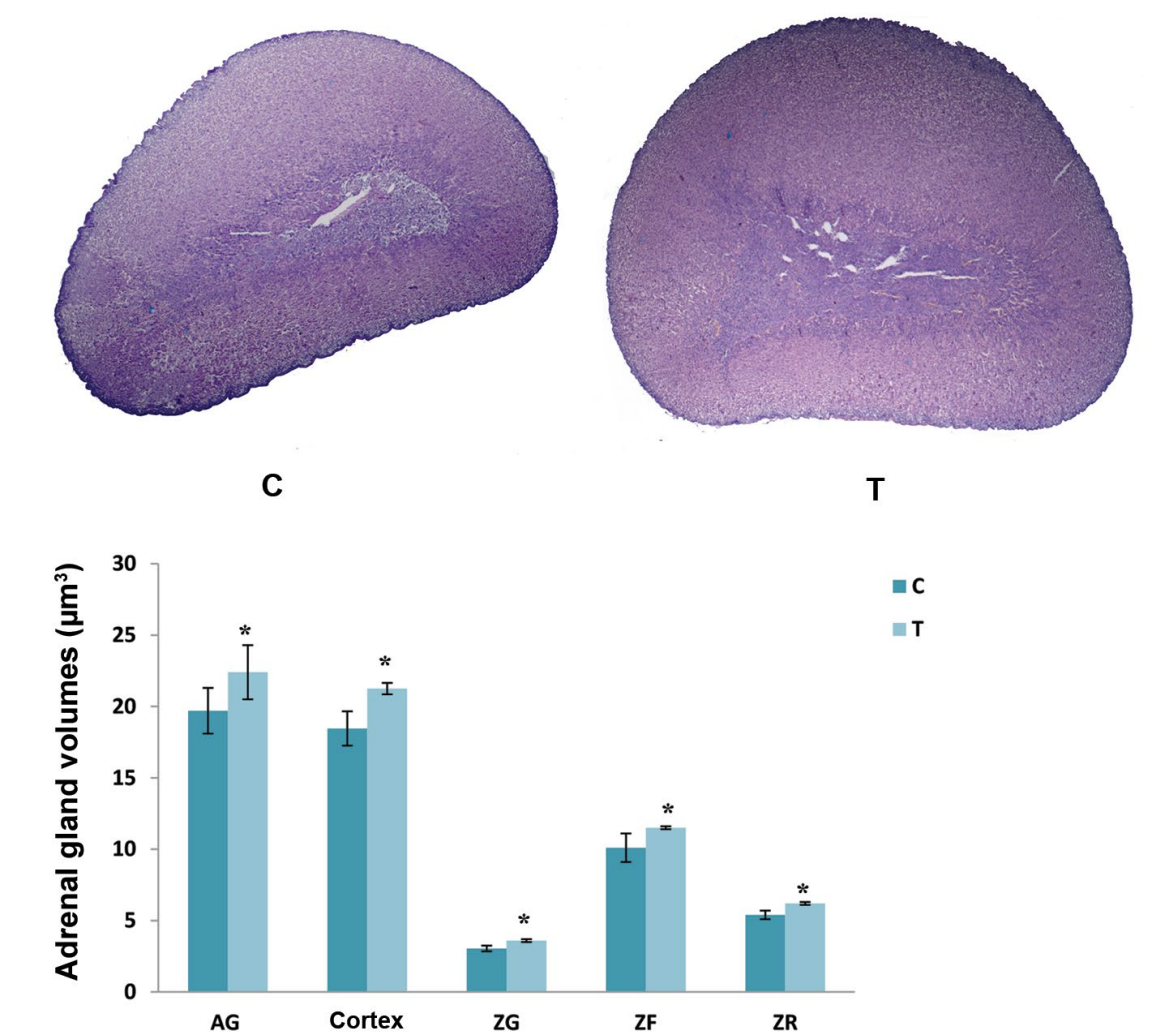
Histological appearance and volumes of the adrenal gland (AG), AG cortex and individual zones within the cortex [zona glomerulosa (ZG), zona fasciculate (ZF) and zona reticularis (ZR)] in the control group (C) and after exposure to the elevated temperature for four days (T). Results are expressed as the mean ± SD. *; P<0.05 vs. control (scale bar: 400 µm).

Stereological measurements and qualitative histological insight after the rat exposure to elevated temperature revealed a significant increase in adrenal gland volume (14%), volume of adrenal gland cortex (15%) and the individual cortical zones (ZG 18%, ZF 15%, ZR 14%), when compared with the adequate control values (Fig.1).

**Fig.2:**
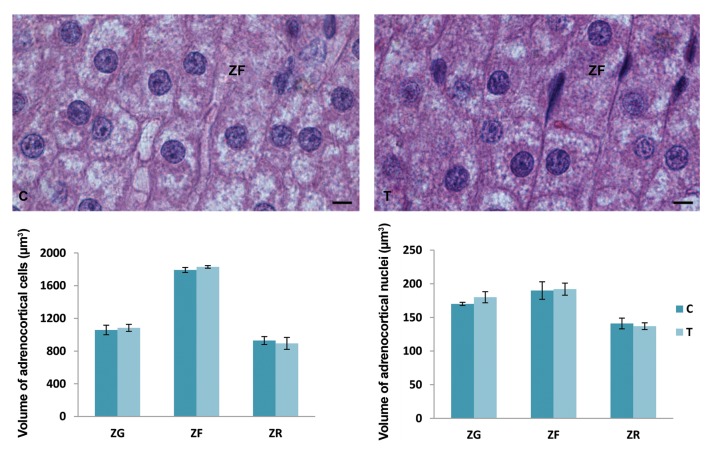
Volumes of the adrenocortical cells and their nuclei in individual zones within the cortex [zona glomerulosa (ZG), zona fasciculate (ZF) and zona reticularis (ZR)] and histological presentation of ZF cells (hematoxylin-eosin staining) in the control group (C) and after exposure to the elevated temperature for four days (T). Results are expressed as mean ± SD (scale bar: 8 µm).

The volume of adrenocortical cells and their nuclei in each zone of the adrenal gland cortex did not significantly change after a four-day exposure to an elevated temperature in comparison with the control values (Fig.2).

**Table 1:** The body mass, as well as the absolute and relative adrenal gland weights, after exposure to the elevated temperature for four days


Experimental group	Body mass (g)	Absolute adrenal gland weight (mg)	Relative adrenal gland weight (mg %)

Control	337.5 ± 26.9	20 ± 1.2	6.9 ± 0.6
Elevated temperature-exposed	270.6 ± 11.7*↓	23.2 ± 1.5*↑	8.6 ± 0.6*↑


Results are expressed as mean ± SD. *; P<0.05 vs. control

**Fig.3:**
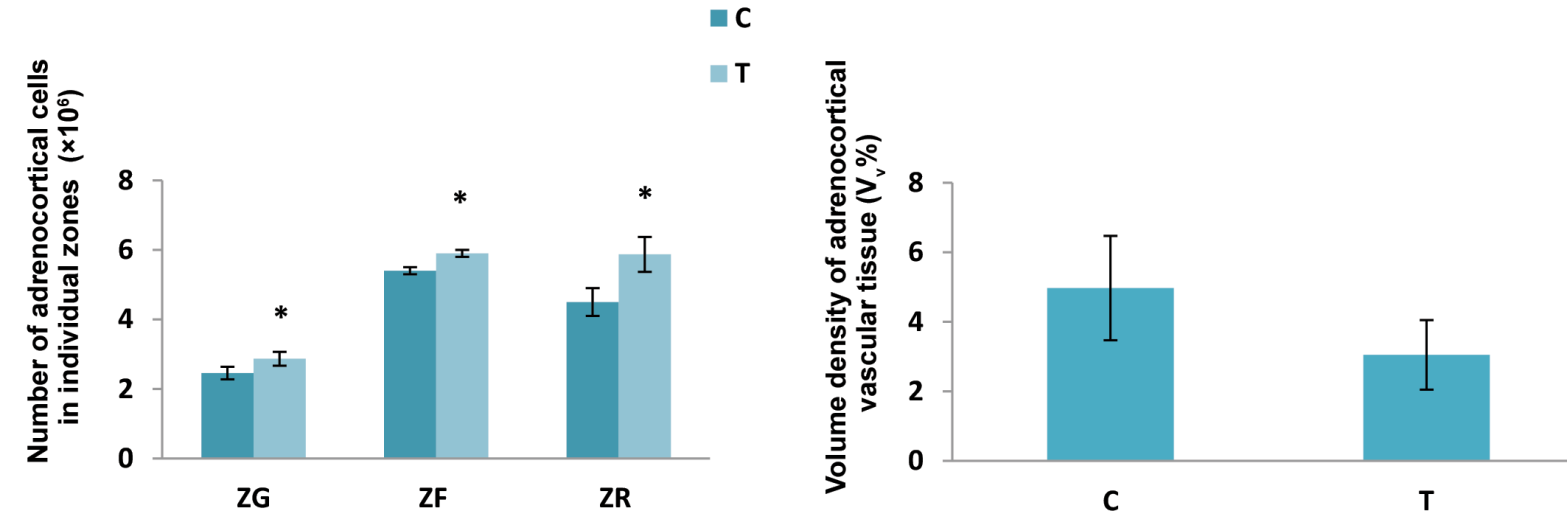
The number of adrenocortical cells in individual adrenocortical zones [zona glomerulosa (ZG), zona fasciculate (ZF), zona reticularis (ZR)] and volume density of vascular tissue in adrenal gland cortex in control animals (C) and after exposure to the elevated temperature for four days (T). Results are expressed as the mean ± SD. *; P<0.05 vs. control.

The elevated temperature exposure for four days caused a significant increase in the number of adrenocortical cells in the ZG, ZF, and ZR of adrenal gland cortex compared with the values established in the control group. Namely, a significant increase in the number of adrenocortical cells in ZG was 17%, 9% in ZF and 30% in ZR. The volume density of vascular tissue in the adrenal cortex did not change under the influence of elevated temperature (Fig.3).

The hormonal analysis showed a substantial increase in the plasma ACTH level by 24%, after exposure to elevated temperature in comparison to the control group. Additionally, 4-day exposure to the elevated temperature led to a significant increase in aldosterone by 16%, while the raise of corticosterone concentration was 25% in comparison to the control values (Table 2).

### Histological analysis

**Fig.4:**
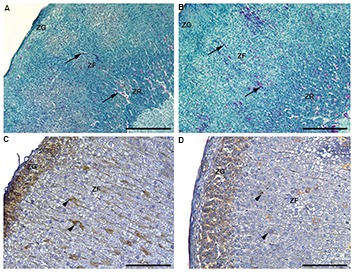
Histological evaluations of the adrenal gland. **A.** The Novelli stained sections of adrenal glands in controls and **B.** After exposure to the elevated temperature for four days. The arrows indicate the blood vessels (scale bar: 200 µm). Immunohistochemical staining of vascular endothelial growth factor (VEGF) in **C.** The adrenal glands in controls and **D.** After exposure to the elevated temperature for four days. Arrow tip indicates VEGF depots (scale bar: 100 µm). ZG; Zona glomerulosa, ZF; Zona fasciculate, and ZR; Zona reticularis.

After the histological examination of hematoxylin/ eosin and Novelli-stained sections, the characteristics of a clear zonation pattern in the adrenal cortex was observed (Fig.4A, B). Outermost situated, sphericallyorganized ZG cells were changed with radially arranged cords of ZF cells and an anastomosing network made of ZR cells that occupied the innermost portion of the cortex. As confirmed after unbiased stereological measurement, only the volume of individual zones differs, while the vascular tissue remained unchanged when the elevated temperature-exposed and control sections were compared.

There were no significant differences in the immunohistochemical appearance and number of VEGF immunopositive cells in the adrenal cortex when the control group and the group exposed to a moderately elevated temperature were compared. VEGF immunostaining was intensive in the cells of ZG in both groups. Moreover, VEGF was expressed in several individual ZF and ZR adrenocortical cells. Cytoplasmic immunopositivity differs among the cells in inner adrenocortical zones: intense immunostaining was noted in some cells, as well as diffuse cytoplasmic immunopositivity. The presence of lipid droplets was also evident in adrenocortical cells (Fig.4C, D).

**Table 2:** The circulating concentration of pituitary adrenocorticotropic hormone (ACTH), as well as adrenocortical hormones, aldosterone, and corticosterone, in controls and after exposure to the elevated temperature for four days


Experimental group	ACTH (pmol/L)	Aldosterone (nmol/L)	Corticosterone (nmol/L)

Control	25.15 ± 0.88	20 ± 1.2	6.9 ± 0.6
Elevated temperature-exposed	31.12 ± 0.57^*^↑	23.2 ± 1.5^*^↑	8.6 ± 0.6^*^↑


Results are expressed as means ± SD. *; P<0.05 vs. control.

## Discussion

Actual trend of global warming, being established during the last 50 years, cannot be explained only by natural cycles, but the anthropogenic influence has also been recognized to possess a significant impact. The most significant consequence of the mentioned trend, an increase in the average annual temperature is particularly pronounced during the summer months in South-Eastern Europe (9). In the region of western Balkans, elevated summer temperatures often last several days in continuity, representing a serious challenge to the homeostasis, and living world actively adjusts to the conditions arising from the environment. The function of the HPA axis follows the seasonal and daily temperature rhythm, while the markedly increased activity of the axis reflects the response to a moderate increase in ambient temperature of 35 ± 1℃ for four days, as the results of this study clearly show. Circulating ACTH level was significantly increased, thus affecting adrenal glands that respond to both structural and functional alterations. It should also be mentioned that decreased bodyweight, as a consequence of 4-day exposure to moderately high ambient temperature, was established. Previous research noticed the reduction of food intake in rats after heat exposure, probably caused by the inhibition of hypothalamic peptidergic circuitry related to food intake and energy balance (21, 22).

Acute short-term heat exposure (38˚C, 60 minutes) leads to a significant elevation in plasma ACTH, representing the response to hyperthermia, as expected (23). The elevated temperature exposure for four days, performed in the previous histological study (11), has provoked more comprehensive changes in pituitary corticotrophs: weak immunopositivity, confirmed by a decrease in the relative fluorescence intensity of the individual ACTH cells, as well as the reduction of the volume density and the increase in the size of these cells. Thus, morphometric parameters and immunofluorescent features pointed that under described experimental conditions, the intensive synthetic and secretory activity of ACTH cells takes place, followed by a significant rise in blood ACTH concentration (11). In oppose to the acute stress episodes, characterized by the fast reestablishment of homeostasis and returning to the basal level, during constant exposure to a moderate increase in ambient temperature, the central brain mechanisms of glucocorticoid feedback inhibition are altered. Inhibitory hippocampal neurons drive towards hypothalamic CRH neurons, exerted via multi-synaptic pathways, is decreased, so CRH neurons are able to constantly stimulate pituitary ACTH cells functioning (24). Moreover, glucocorticoid feedback inhibition from the amygdala to hypothalamic PVN has been attenuated in chronic stress conditions (25). The elevated temperature exposure for four days obviously activates different pattern of ACTH cell response to the applied chronic stimulus, associated with desensitization of the ACTH cells, that led to constant hormonal levels and consequently to a significant increase in ACTH level in circulation (11).

ACTH is a trophic hormone, which, through the activation of the melanocortin 2 receptor, controls the proliferation of adrenocortical cells and promotes steroidogenesis (26). In the present study, the absolute and relative weight gain of the adrenal gland was clearly shown. The adrenals were stimulated after four days of exposure to elevated temperature. A significant increase in the adrenocortical volume was the consequence of a marked increase in the volume of each adrenocortical zone separately. The unchanged volumes of individual cells and their nuclei, in all cortical zones, were observed in parallel with a significantly increased number of adrenocortical cells. Taking into consideration that the volume density of vascular tissues in the entire cortex was not significantly altered, it could be concluded that the increase in the proliferation rate of adrenocortical cells was an adaptive response to meet the increased physiological demands under the given conditions. Temporal prolongations of the temperature stress for four days, which resulted in the adrenal gland hypertrophy, in fact, has enabled baseline glucocorticoid hypersecretion for an extended period. Intolerance to heat exposure is associated with HPA axis impairment, followed by decreased plasma corticosterone and ACTH levels (27). The intensified process of steroidogenesis after heat exposure, and consequently elevated corticosterone level in circulation, reported here, was accompanied by numerous morphological changes at the level of electron microscopy of adrenocortical cells. Close apposition of specific lipid droplets to the cytoplasmic face of the cell membrane, increased mitochondrial volume density, the close morphological relationship between lipid droplets and mitochondria together with pronounced smooth endoplasmic reticulum, pointed that functional engagement of alleged organelles was needed for intensive steroid hormone synthesis (23). Generally, some lipid droplets contain cholesterol as a steroid hormone precursor, others contain hormone itself or its immediate precursors, as the forms of steroid hormones storage, and release them into the capillary network (28). This is in line with the reports of Nussdorfer (29), who found that the long-term trophic effect of ACTH is involved not only in an increase of the adrenal mass but also in the stimulation of the ZF and organelles involved in the steroidogenic activity of ZF cells. A stimulatory effect of ACTH on adrenocortical ZF cells was characterized by hypertrophy and enhancement of the steroidogenic enzymeactivity (30, 31)

Additionally, ACTH stimulates the proliferation of the adrenocortical cells, mostly in ZG and outer ZF region, and enhances the centripetal migration of newly-formed cells and their accumulation in ZR (32). Consequently, the significantly increased number of ZR cells and ZR volume were established after four days of the elevated temperature exposure in our experiment

VEGF is the major mediator of angiogenesis and a potent inducer of endothelial fenestration during vascularization of the adrenal gland (33). The ZG, as the place with a massively developed vasculature network, is characterized by VEGF presence in the cytoplasm of all cells, as presented. As in the ZG, the elevated temperature did not have any significant effect on the presence of VEGF-positive adrenocortical cells in ZF and ZR. In parallel, the same percentage of vascular tissue was measured in both examined groups. Thus, the immunohistochemical appearance of the VEGF-positive adrenocortical cells and determined vascular volume density pointed out that ambient temperature of 35 ± 1℃ did not significantly influence the circulatory aspect of the adrenal gland. The activated physiological mechanisms caused the blood flow is increased peripherally, in the skin, in order to allow greater heat dissipation in given experimental conditions.

Some earlier reports, elaborating the effect of moderately high ambient temperature (35 ± 1℃), suggesting the decreased serum corticosterone level after 24-48 hours of the heat exposure, through a feedback mechanism resulting from the acute elevated temperature exposure of rats within the first day (15). Such a decrease is followed by an elevation of a serum corticosterone level again (34), which is consistent with our earlier results. Furthermore, normalization or even a decrease in the activity of pituitary-adrenocortical axis after the long-term exposure (28 days) to a moderately high ambient temperature has been reported, suggesting an acclimation of the organism during exposure to a persistent environmental stressor (35).

Our findings of the increased volume of ZG, increased number of the cells in this zone, and increased blood aldosterone concentration probably result from ACTH stimulation in the elevated temperature-exposed group. It was found that although ACTH primarily regulates glucocorticoid production in ZF cells, this pituitary hormone can also provoke the ZG cells activity (36). Some reports found that chronic ACTH and cAMP treatment might induce hyperplasia and mitotic activity in ZG cells (37). Additionally, the strong stimulus for aldosterone synthesis during the elevated temperature exposure and increased aldosterone blood level reported here, supposedly stem from the sodium depletion, which presumed to occur together with the dehydration, in order to preserve mineral homeostasis (7). Increased synthetic and secretory activity of the ZG cells under elevated temperature regime showed the ultrastructural level, as an increased number of mitochondria and lipid depletion (23). Increased plasma renin and aldosterone concentrations were also found after five days of the continuous heat exposure to a moderate increase in ambient temperature (38). According to Saini et al. (39) elevated aldosterone concentration in men, as a result of increased activity of the renin-angiotensin system, could be a compensatory mechanism against mineral and water losses, which occurs after six days of passive heat exposure. Other studies also showed that under stress conditions, both ACTH and VP secretion increase, which stimulates aldosterone release (31).

## Conclusion

The presented results could provide the additional example of the HPA axis capacity to respond to prolonged stimulation of a moderate increase in ambient temperature, enabling the body to adapt to the daily temperature changes and, consequently, successful acclimatize an organism. Future studies will be directed to the elucidation of the concrete mechanisms underlying the changes in the HPA axis activity upon elevation in an ambient temperature.
